# Atrial Fibrosis in Atrial Fibrillation: Mechanistic Insights, Diagnostic Challenges, and Emerging Therapeutic Targets

**DOI:** 10.3390/ijms26010209

**Published:** 2024-12-30

**Authors:** Paschalis Karakasis, Panagiotis Theofilis, Panayotis K. Vlachakis, Panagiotis Korantzopoulos, Dimitrios Patoulias, Antonios P. Antoniadis, Nikolaos Fragakis

**Affiliations:** 1 Second Department of Cardiology, Hippokration General Hospital, Aristotle University of Thessaloniki, 54642 Thessaloniki, Greece; aantoniadis@gmail.com (A.P.A.); fragakis.nikos@googlemail.com (N.F.); 2 First Cardiology Department, School of Medicine, Hippokration General Hospital, National and Kapodistrian University of Athens, 11527 Athens, Greece; panos.theofilis@hotmail.com (P.T.); vlachakispanag@gmail.com (P.K.V.); 3 First Department of Cardiology, School of Health Sciences, Faculty of Medicine, University of Ioannina, 45500 Ioannina, Greece; pkorantz@uoi.gr; 4 Second Propedeutic Department of Internal Medicine, Faculty of Medicine, School of Health Sciences Aristotle, University of Thessaloniki, 54642 Thessaloniki, Greece; dipatoulias@gmail.com

**Keywords:** atrial fibrosis, atrial fibrillation, arrhythmogenic mechanism, inflammation, protease-activated receptor inhibitors, SGLT2 inhibitors, GLP1 receptor agonists

## Abstract

Atrial fibrosis is a hallmark of atrial cardiomyopathy and plays a pivotal role in the pathogenesis of atrial fibrillation (AF), contributing to its onset and progression. The mechanisms underlying atrial fibrosis are multifaceted, involving stretch-induced fibroblast activation, oxidative stress, inflammation, and coagulation pathways. Variations in fibrosis types—reactive and replacement fibrosis—are influenced by patient-specific factors such as age, sex, and comorbidities, complicating therapeutic approaches. The heterogeneity of fibrosis leads to distinct electrophysiological abnormalities that promote AF via reentrant activity and enhanced automaticity mechanisms. Despite advancements in imaging, such as late gadolinium enhancement CMR and electroanatomical mapping, challenges in accurately quantifying fibrosis persist. Emerging therapeutic strategies include antifibrotic agents targeting the renin–angiotensin–aldosterone system, novel pathways like TGF-β signaling, and cardio-metabolic drugs like SGLT2 inhibitors and GLP-1 receptor agonists. Innovative interventions, including microRNA modulation and lipid nanoparticle-based therapies, show promise but require validation. Knowledge gaps remain in correlating clinical outcomes with fibrosis patterns and optimizing diagnostic tools. Future research should focus on precise phenotyping, integrating advanced imaging with molecular biomarkers, and conducting robust trials to evaluate antifibrotic therapies’ efficacy in reducing AF burden and related complications.

## 1. Introduction

Atrial fibrosis is increasingly recognized as a critical prognostic factor in the development of atrial fibrillation (AF) and associated complications, including stroke [1,2,3,4,5]. It is strongly linked to various cardiovascular comorbidities of AF, such as heart failure and valvular disorders, and constitutes a central component of atrial cardiomyopathy [6,7,8]. Atrial cardiomyopathy encompasses electrical, mechanical, and structural alterations of the atria, which collectively lead to clinically significant manifestations [6,7,8]. Both experimental and clinical studies highlight a bidirectional relationship between AF and atrial fibrosis, wherein AF can induce fibrosis, and fibrotic remodeling, in turn, exacerbates the risk and progression of AF [9]. The mechanisms driving atrial fibrosis are multifaceted and dynamic and exhibit considerable variability among individuals, reflecting the diverse clinical conditions underlying its pathogenesis [10,11,12].

**Figure 1:**
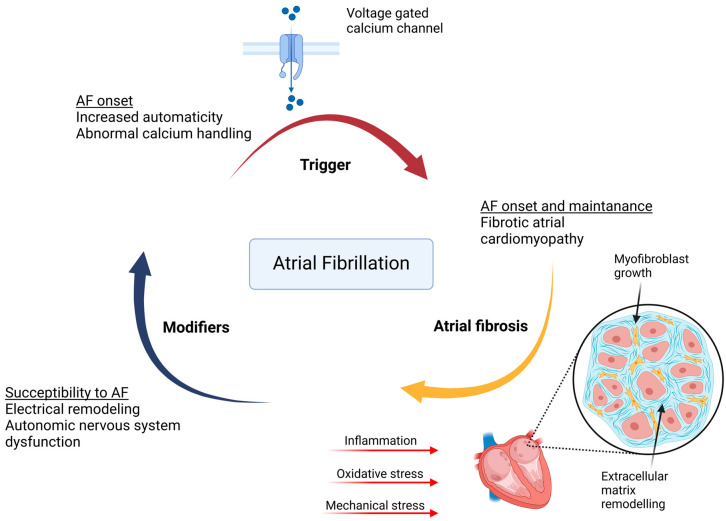
Pathogenesis of Atrial Fibrillation. The figure summarizes the multifactorial mechanisms driving atrial fibrillation (AF) development and progression. Key contributors include atrial fibrosis, stretch-induced fibroblast activation, oxidative stress, inflammation, and coagulation pathway activation. Reactive and replacement fibrosis alters atrial tissue architecture, contributing to conduction abnormalities through re-entry circuits and enhanced automaticity. Oxidative stress and inflammation exacerbate atrial remodeling, while coagulation pathways further enhance the arrhythmogenic substrate. These interconnected processes lead to electrical and structural remodeling, creating a vicious cycle that sustains AF.

The pathophysiology of atrial fibrosis involves several interconnected processes, including stretch-induced fibroblast activation, the generation of reactive oxygen species (ROS), localized and systemic inflammatory responses, activation of coagulation pathways, and fibrofatty infiltrations (Figure 1) [13,14]. Although fibroblasts and their differentiated form, myofibroblasts, are the primary cellular sources of collagen fiber production, the initiating stimuli often originate from cardiomyocytes, adipocytes, or immune cells [10,11,12]. These cell types respond to clinical insults by releasing paracrine signals that stimulate fibroblast activity [10,11,12]. Atrial fibrosis manifests in distinct patterns depending on the underlying stimuli. Reactive fibrosis, characterized by perimysial and endomysial collagen deposition, arises from chronic moderate stress and occurs without cardiomyocyte death [15]. In contrast, replacement fibrosis, associated with cardiomyocyte loss, features extensive collagen deposition, often forming patchy networks [14]. While multiple fibrosis types may coexist within the atria, endomysial fibrosis appears particularly implicated in the pathophysiology of AF [16,17].

The heterogeneity in fibrotic patterns and their underlying mechanisms presents significant challenges in identifying molecular pathways driving atrial fibrosis and developing effective therapeutic strategies [18]. Recent advances in phenotyping atrial fibrosis have enhanced our understanding of its role in atrial conduction abnormalities and may facilitate the identification of disease-specific mechanisms [19]. This progress offers opportunities to explore novel therapeutic targets and biomarkers, enabling more precise, individualized antifibrotic treatments. This review provides a comprehensive analysis of how different fibrosis patterns disrupt atrial conduction, explores the electrophysiological consequences of fibrosis at the organ level, and critically evaluates current diagnostic methods for quantifying fibrosis. Additionally, emerging therapeutic approaches targeting inflammatory and coagulation processes are discussed alongside novel biotechnological interventions. Finally, we highlight existing knowledge gaps and propose future research directions to address unresolved challenges in this field.

## 2. Electrophysiological Implications of Atrial Fibrosis

### 2.1. Atrial Fibrosis and Arrhythmogenicity

The two primary pro-arrhythmic mechanisms contributing to the initiation and maintenance of AF are reentrant activity and enhanced automaticity [20]. Atrial fibrosis plays a critical role in enhancing the propensity for both mechanisms, albeit through processes operating at different spatial scales.

Re-entry involves the continuous propagation of fibrillatory waves through atrial tissue. This mechanism can manifest in a hierarchical pattern, where one or more localized circuits drive the arrhythmia, or a non-hierarchical pattern, characterized by the chaotic propagation of multiple wavelets without a dominant source of activity [9]. Localized re-entry can further be categorized into anatomical and functional types. Anatomical re-entry occurs when waves circulate around a fixed, unexcitable obstacle, while functional re-entry presents as spiral wave patterns in a uniformly excitable substrate [21,22,23]. Fibrous tissue can serve as the unexcitable core of an anatomical re-entry circuit or establish the center of rotation for a functional re-entry pattern. However, fibrosis can also destabilize functional spiral waves, disrupting their organization. Stable re-entrant circuits may produce waves that fragment due to obstacles such as fibrous tissue or areas with prolonged refractoriness, resulting in more chaotic conduction patterns at sites distant from the primary circuit [24,25]. The persistence of fibrillation increases with the complexity of conduction patterns, while termination becomes more likely as the number of wavefronts decreases [9]. Animal studies have consistently demonstrated that fibrosis correlates with heightened complexity of fibrillatory conduction and increased AF persistence [26,27].

Enhanced automaticity, driven by abnormalities in cardiomyocyte electrophysiology, constitutes another key pro-arrhythmic mechanism [11]. Dysregulated calcium handling within cardiomyocytes can trigger after-depolarizations following a normal action potential. In electrically well-coupled tissue, depolarizing currents generated by a single cardiomyocyte dissipate rapidly, failing to reach the action potential threshold in neighboring cells [28]. However, the likelihood of after-depolarizations generating a propagated action potential increases when synchronization occurs over a larger area [29]. Poor electrical coupling, often associated with fibrosis, facilitates this synchronization [30]. Fibrosis-induced electrical uncoupling is particularly conducive to enhanced automaticity in regions with a gradient of electrical connectivity, transitioning from areas of poor coupling to normal myocardium [31].

Animal research in transgenic mouse models overexpressing TGFβ1, which induces selective atrial fibrosis, exhibits increased conduction heterogeneity—consistent with re-entry—and heightened enhanced automaticity, underscoring the integral role of fibrosis in exacerbating the arrhythmic substrate through diverse electrophysiological mechanisms [32,33]. In summary, fibrosis promotes both reentrant activity and enhanced automaticity, amplifying the basic mechanisms underlying AF.

### 2.2. Association Between Fibrosis and Tissue Architecture

The distinct histological patterns of fibrosis described earlier have diverse effects on electrical propagation within cardiac tissue [26]. These effects are not primarily determined by the overall extent of fibrosis but rather by the disruption of specific electrical connections caused by the presence of fibrotic tissue. Although this alteration cannot currently be assessed directly, it is generally presumed to have an inverse relationship with the degree of fibrosis [26].

At the microscopic scale, cardiomyocytes connect end-to-end through intercalated discs, which contain large gap junctional plaques, facilitating robust electrical coupling. In contrast, side-to-side connections are fewer and characterized by smaller gap junctional plaques [34]. Cardiomyocytes are aligned in longitudinally oriented strands, resulting in anisotropic conduction, where electrical signals propagate faster along the longitudinal axis than in the transverse direction. Transverse delays in activation between adjacent strands can occur, and these delays become more pronounced as transverse connections diminish [35,36]. Endomysial fibrosis, also referred to as interstitial or microfibrosis, disrupts transverse connections by thickening collagen septa. This phenomenon, commonly associated with aging or AF-induced remodeling, is strongly linked to discontinuous conduction and increased AF complexity [37]. However, the quantitative relationship between endomysial fibrosis thickness and the loss of transverse connectivity remains unclear. Interestingly, the reduced density of side-to-side connections in fibrotic tissue may paradoxically enhance longitudinal conduction velocity, as demonstrated in vitro using human atrial trabeculae [38]. This finding could explain why certain studies have reported shorter atrial conduction times in patients with increased fibrosis [39].

At the macroscopic level, cardiomyocytes are organized into bundles separated by perimysial fibrous tissue [40]. These bundles exhibit a branching architecture, with individual strands occasionally crossing between adjacent bundles [40]. Transverse electrical propagation at this scale likely depends on both longitudinal and transverse gap junctions. However, detailed data on the spatial distribution of these branching points between bundles, which are critical for transverse conduction, is limited. For example, histological studies of Bachmann’s bundle, the primary interatrial conduction pathway, reveal a preferential fiber orientation and transverse connections between bundles spaced up to 0.5 mm apart [36]. The architecture of myocyte bundles varies regionally within the atria [41]. In the atrial free walls, for instance, the endocardial side forms an extensive branching network overlain by a less organized epicardial layer [42].

The atrial wall’s layered structure, featuring regions of differing fiber orientations (e.g., longitudinal epicardial fibers and circumferential endocardial fibers), creates transitional zones, such as those near the pulmonary veins, that are susceptible to conduction delays or block [43,44]. Despite the critical role of transverse connectivity in maintaining conduction integrity, the regional variability of this connectivity and its modulation by different types of fibrosis remain poorly understood.

### 2.3. Impact of Atrial Fibrosis on Electrical Conduction in Clinical and Preclinical Studies

The relationship between atrial fibrosis and electrical conduction has been extensively studied using large-animal models. A pivotal study from 1999 compared two canine models: one with rapid atrial pacing (RAP), simulating AF in the absence of structural heart disease, and another with ventricular tachypacing-induced congestive heart failure (CHF) [45]. Unlike the RAP model, which did not exhibit increased overall cardiac fibrosis, the CHF model demonstrated substantial fibrotic tissue deposition in the left atrium, indicative of reparative fibrosis replacing necrotic cardiomyocytes [46,47]. This patchy fibrosis facilitates re-entrant circuits by creating conduction pathways around areas of fibrotic tissue. During slow pacing, increased conduction heterogeneity was observed in the CHF model but was absent in the RAP model [45]. Although both models exhibited enhanced AF stability, the RAP model was associated with more intricate fibrillation patterns and reduced responsiveness to the anti-arrhythmic drug dofetilide [48]. This suggests differences in the underlying arrhythmogenic mechanisms between the models. Notably, atrial fibrosis and conduction abnormalities persisted even after recovery from CHF, highlighting the largely irreversible and critical role of atrial fibrosis as a pro-arrhythmic substrate in this model [49].

The effects of AF on ventricular response and structural remodeling have been extensively studied [50,51]. Notably, structural changes are observed not only in the context of AF with rapid ventricular response but also in cases where ventricular rates are controlled [52]. In a goat model of AF, RAP without increased ventricular rates induced a slow, progressive structural remodeling process over several months [53,54]. This remodeling was associated with more complex fibrillation dynamics, characterized by an increased number of smaller, simultaneously propagating wavefronts and a complete loss of anti-arrhythmic drug efficacy [53,54]. After six months of sustained AF, the overall area of fibrotic tissue remained unchanged, but fibrosis within the outermost millimeter of the atrial wall increased, resulting in impaired transverse electrical conduction [53,54]. Clinical observations align with these findings. In patients undergoing cardiac surgery, fibrosis is consistently more pronounced in the subepicardial layer compared to the endocardial bundle network [55]. Experimental and computational modeling studies have further elucidated the impact of this pattern of fibrosis, demonstrating increased endocardial–epicardial desynchrony during AF [56,57]. Key contributors to this phenomenon include reduced connectivity between atrial wall layers, preferential conduction block in the subepicardial layer, and sharp differences in myocardial bundle orientation between the endocardium and epicardium [58,59,60]. These factors collectively promote endocardial–epicardial dissociation, the emergence of breakthrough waves, and increased fibrillation complexity [58,59,60]. This interplay between structural remodeling and electrical conduction underscores the role of localized fibrotic changes in exacerbating AF dynamics and sustaining arrhythmia.

Understanding the relationship between fibrosis and AF in patients is complicated by the presence of diverse underlying risk factors that often coexist. These factors not only contribute to fibrosis but also induce additional structural alterations that affect electrical conduction. Such changes include cardiomyocyte hypertrophy [61], fatty infiltration [62,63], altered expression of connexins [64], and, potentially, the formation of aberrant electrical connections between cardiomyocytes and fibroblasts [65,66]. Evidence regarding the association between AF and fibrosis has been mixed. For instance, one study demonstrated that mitral valve disease—a significant risk factor for AF—is associated with increased atrial fibrosis [67], while AF itself did not independently correlate with fibrosis [68]. Conversely, another study found higher levels of fibrosis in patients with AF compared to age-matched controls without AF, with the extent of fibrosis being greater in individuals with permanent AF than in those with paroxysmal AF [69].

A comprehensive study published in 2023 systematically evaluated the relationships between age, sex, comorbidities, and atrial fibrosis [16]. The findings identified heart failure, female sex, and a history of AF as the primary clinical factors associated with fibrosis in the left atrium. Notably, the contribution of age to atrial fibrosis was minimal [16]. An important discovery from this study was the association between persistent AF and endomysial fibrosis in the left atrium, while no relationship was observed with the overall connective tissue content [16]. This aligns with earlier research suggesting that conduction abnormalities in the atria are linked specifically to endomysial fibrosis rather than to total fibrosis burden [17]. Another investigation reported no correlation between fibrosis and propagation properties; however, it should be noted that electrical conduction in this study was evaluated exclusively during sinus rhythm, potentially limiting the findings [70].

Conduction abnormalities caused by fibrosis may only become evident under specific conditions, such as during short coupling intervals or fibrillatory conduction. Under these circumstances, the incomplete recovery of the sodium current exacerbates source-to-sink mismatches, impairing electrical propagation [71,72]. Evidence for this relationship comes from a study that directly correlated fibrotic tissue with electrical conduction, revealing that in the left atrium, regions with thicker fibrotic strands exhibit longer activation times during extra-stimulation compared to areas with thinner strands [73]. Another investigation employing late gadolinium enhancement cardiovascular magnetic resonance (LGE-CMR) to detect fibrosis demonstrated that in human right atria perfused with pinacidil (a drug that shortens action potential duration), atrial fibrillation was maintained by intramural re-entry circuits anchored to atrial bundles insulated by fibrotic tissue [74]. However, the extent to which this behavior reflects human AF at more physiological cycle lengths remains unclear. Epicardial mapping studies in humans have identified key conduction patterns in advanced AF, including discontinuous conduction, longitudinal dissociation characterized by narrower and more numerous fibrillation waves [75], multiple pivot points [76], and endocardial–epicardial dissociation [77,78]. While these studies did not directly compare electrical conduction to underlying tissue characteristics, the observed conduction patterns are likely attributable, at least in part, to endomysial atrial fibrosis. Table 1 summarizes the studies investigating the impact of atrial fibrosis on electrical conduction.

## 3. Imaging Modalities and Markers of Atrial Fibrosis

### 3.1. Imaging Methods

To elucidate the pathophysiological role and prognostic significance of atrial fibrosis in cardiovascular diseases, there is a critical need to identify clinical indicators or surrogate markers of this condition. While imaging techniques such as echocardiography and cardiac CT cannot directly detect atrial fibrosis, they do provide measurements of atrial volume, size, and function [8]. Advanced echocardiographic parameters, such as tissue strain, have recently gained validation and correlate with atrial mechanical function [79]. Histological analyses of biopsy specimens from heart transplant recipients [80] and patients with pulmonary arterial hypertension [81] have confirmed that atrial fibrosis reduces mechanical function by impairing atrial compliance. These imaging modalities may thus offer indirect insights into the quantification of atrial fibrosis.

Over the past decade, late gadolinium enhancement LGE-CMR has emerged as a tool for imaging and quantifying atrial fibrosis [79]. However, its utility is constrained by limited robustness and reproducibility due to the lack of standardized acquisition and processing protocols, as well as the challenges posed by the thin structure of the left atrial wall and the relatively low image resolution [79]. For example, substantial regional and global variability in LGE-CMR findings has been observed, indicating significant spatial heterogeneity of atrial fibrosis [82]. Traditional signal thresholding techniques in LGE-CMR primarily detect focal scarring, whereas experimental and clinical evidence suggests that atrial fibrosis associated with atrial fibrillation or rapid ventricular pacing often exhibits a more homogeneous distribution [83]. Furthermore, diffuse endomysial fibrosis, which plays a critical role in conduction disturbances, may be underrepresented or missed entirely. Histological assessments have shown only a moderate correlation between endomysial fibrosis and total connective tissue content, implying that while LGE-CMR signals can partially reflect total connective tissue, they may not reliably capture the nuances of endomysial fibrosis [16].

Studies in animal models, such as dogs subjected to rapid ventricular pacing, have quantified increases in atrial fibrosis using both LGE-CMR and histological analysis, but the high interobserver variability in LGE-CMR interpretation has limited its reliability [83]. Electroanatomical catheter mapping with high-density techniques is another method used to identify low-voltage areas (LVAs) in the atria, which are often considered surrogates for localized atrial fibrosis [17,42].

Recent studies have highlighted its utility in characterizing atrial low-voltage regions and guiding ablation strategies. A study by Compagnucci et al. [84] demonstrated the efficacy of microbipolar and bipolar high-density mapping in refining voltage cutoffs for identifying atrial low-voltage areas. The adjusted microbipolar voltage cutoffs (0.71–1.69 mV) correlated with bipolar voltage thresholds (0.16–0.31 mV) and improved substrate characterization during ablation procedures for persistent AF [84]. Moreover, this study compared very-high-power short-duration (vHPSD) ablation with standard-power ablation for posterior wall ablation (PWA) plus pulmonary vein isolation (PVI) [84]. The vHPSD approach was associated with shorter procedural and fluoroscopy times and a trend toward superior efficacy in reducing recurrent AF without compromising safety [84]. These findings underscore the potential of integrating advanced electroanatomical mapping with refined voltage thresholds to enhance the identification and treatment of atrial fibrosis during ablation [84].

Experimental and clinical studies have established a relationship between endomysial fibrosis and conduction heterogeneity, which manifests as low electrogram voltage. Moreover, LVAs have been associated with adverse outcomes such as atrial fibrillation recurrence [85,86] and increased risks of stroke or mortality [87]. However, LVAs do not consistently align with histological or LGE-CMR findings, and validation studies have demonstrated significant discrepancies [82,83].

Despite these limitations, some LGE-CMR studies have provided meaningful pathophysiological insights, such as the preferential localization of atrial re-entrant circuits at the interface between fibrotic and non-fibrotic regions [88]. Nevertheless, neither LGE-CMR nor LVAs provide direct measurements of atrial fibrosis. Various other factors can influence increased LGE signal intensity and the presence of low-voltage electrograms. Therefore, referring to these phenomena as “atrial fibrosis” is imprecise and should be approached with caution. Table 2 summarizes the studies evaluating imaging methods for diagnosing and characterizing atrial fibrosis.

### 3.2. Blood Biomarkers

Numerous blood biomarkers are currently under investigation as prognostic indicators in patients with AF and as potential surrogate measures for the extent of atrial fibrosis [8]. While these biomarkers have been extensively studied in the context of AF, their ability to specifically reflect atrial fibrosis in the absence of AF remains less well explored [90,91,92,93,94]. Among the biomarkers investigated—such as natriuretic peptides (BNP and ANP), cardiac troponin T, soluble interleukin-1 receptor-like 1 (ST2), TIMP1, adhesion molecules (e.g., ICAM1 and VCAM), pro-inflammatory cytokines (e.g., CCL2), and protease-activated receptors (PAR1, PAR2, and PAR4)—most have been evaluated in the context of both AF and atrial fibrosis [90,95,96]. This overlap underscores the limited specificity of these biomarkers in distinguishing atrial fibrosis independent of AF.

Bone morphogenetic protein 10 (BMP10) has emerged as an atrial-specific biomarker predominantly expressed in the right atrium and released into the circulation during the progression of atrial pathologies [97,98]. Evidence from multiple studies has established a correlation between plasma BMP10 levels and the recurrence of AF following AF ablation procedures [97,98]. Notably, BMP10 has also been independently associated with an elevated risk of ischemic stroke in patients with AF, regardless of anticoagulation therapy [99]. Furthermore, findings from a cohort study in individuals with AF revealed significant associations between plasma BMP10 concentrations and both all-cause mortality and adverse cardiovascular events [100]. Crucially, data from the European CATCH ME consortium, which involved histological analysis of patients undergoing cardiac surgery, demonstrated an independent relationship between plasma BMP10 levels and both postoperative AF risk and the severity of endomysial fibrosis in left atrial tissue [101]. These findings may elucidate the link between elevated BMP10 levels and the recurrence of AF following ablation or the incidence of stroke.

## 4. Therapeutic Targeting of Atrial Fibrosis

Dissolving pre-existing collagen deposits within atrial tissue remains a formidable challenge. However, pharmacological interventions may offer the potential to prevent the progression of atrial fibrosis associated with underlying cardiac conditions [102,103,104,105,106]. Experimental evidence suggests that targeting the renin–angiotensin–aldosterone system (RAAS) using angiotensin-converting enzyme (ACE) inhibitors, angiotensin II receptor blockers, or mineralocorticoid receptor antagonists may delay or prevent the onset of atrial fibrosis [103,104,105,107]. Despite these findings, clinical studies have primarily focused on the impact of such treatments on AF occurrence rather than directly evaluating their effects on atrial fibrosis itself.

The randomized, placebo-controlled ANTIPAF-AFNET [108] trial provided further insight, demonstrating limited efficacy of angiotensin II receptor blockers in patients with AF without significant underlying cardiac disease. Moreover, pathways such as nuclear factor-κB, NADPH oxidase, lysyl oxidase homolog 2, and the TGFβ1–SMAD2/3 signaling cascade have been implicated in angiotensin II-induced atrial fibrosis [109,110,111,112]. Experimental studies indicate that inhibition of these pathways may mitigate fibrosis development and reduce AF incidence.

Another category of agents with potential antifibrotic effects includes sodium-glucose co-transporter 2 (SGLT2) inhibitors and glucagon-like peptide-1 receptor agonists (GLP-1RAs). These therapies have demonstrated remarkable cardio–renal–metabolic benefits, with emerging evidence indicating their effectiveness in various cardiovascular conditions, including AF incidence or recurrence, irrespective of baseline diabetes status [113,114,115,116,117,118,119,120,121,122,123,124,125,126,127,128,129,130,131,132].

SGLT2 inhibitors mitigate atrial fibrosis through several mechanisms. These agents reduce oxidative stress by improving mitochondrial function and decreasing ROS production [133,134], thereby attenuating pro-fibrotic signaling pathways such as the TGF-β/SMAD axis [135]. Additionally, SGLT2 inhibitors exhibit anti-inflammatory properties, reducing circulating levels of cytokines like IL-6 and TNF-α, which are key contributors to cardiac fibroblast activation and extracellular matrix (ECM) remodeling [136,137,138]. Improved myocardial energy metabolism, facilitated by enhanced ketone body utilization, alleviates cellular stress, while natriuretic and diuretic effects decrease atrial wall stretch and pressure, further reducing mechanical stress-induced fibrosis [138,139,140,141].

GLP-1RAs directly counteract atrial fibrosis by modulating cardiac fibroblast activity through GLP-1 receptor activation [142,143]. This suppresses pro-fibrotic pathways, including TGF-β and connective tissue growth factor (CTGF), and reduces ECM deposition [142,143]. Additionally, GLP-1RAs significantly lower systemic and local inflammation by attenuating macrophage infiltration and pro-inflammatory cytokine levels [144,145]. By enhancing glucose uptake and fatty acid oxidation, these agents optimize cardiac metabolism, reducing metabolic stress that contributes to fibrotic remodeling [146]. GLP-1RAs also bolster antioxidant defenses, diminishing oxidative stress and its fibrotic consequences [147]. Indirectly, they alleviate cardiac hypertrophy, lessening mechanical strain on atrial tissue and further mitigating fibrosis [148,149].

SGLT2 inhibitors and GLP-1RAs could potentially synergistically attenuate atrial fibrosis through complementary pathways. Both drug classes reduce oxidative stress, inflammation, and neurohormonal activation while also improving endothelial function and metabolic efficiency [139,140,141,144,145,150]. Their shared ability to decrease levels of angiotensin II and aldosterone reduces pro-fibrotic signaling and atrial remodeling [151,152]. Furthermore, SGLT2 inhibitors’ diuretic effects [153] and GLP-1RAs’ influence on cardiac hypertrophy [148,149] alleviate atrial wall stress, addressing mechanical drivers of fibrosis. By targeting multiple facets of fibrogenesis, the combination of these therapies provides a comprehensive approach to preventing or reversing atrial fibrosis, particularly in populations at high cardiovascular risk. Of course, it should be mentioned that future studies are needed to determine whether the pathophysiological basis of their synergistic effects translates into a documented reduction in atrial fibrosis.

Additionally, endothelin 1 signaling has been shown to drive atrial remodeling in spontaneously hypertensive rats [154]. Treatment with macitentan, an endothelin receptor antagonist, reduced atrial endothelin 1 levels and suppressed pacing-induced increases in pro-endothelin 1 mRNA within atrial tissue slices. Macitentan also attenuated atrial pro-inflammatory signaling, although its effects on calcium-regulating proteins, hypertrophy markers, and fibrosis indicators were minimal [154].

Protease-activated receptors 1 and 2 (PAR1 and PAR2) have been identified as potential therapeutic targets for the prevention of atrial fibrosis. However, experimental evidence indicates that the effects of coagulation factors on PAR signaling are highly context-dependent, varying across cell types and experimental systems [95,96]. For instance, coagulation factor Xa did not influence the expression of PAR1, PAR2, or PAR4 in the HL-1 mouse atrial cardiomyocyte cell line [95,96,155]. In contrast, it was shown to upregulate PAR1 expression in human atrial tissue cultures and fibroblasts [155]. Consistent with these findings, treatment with nadroparin, an anticoagulant targeting coagulation factors Xa and IIa, demonstrated partial efficacy in preventing atrial fibrosis and mitigating the development of complex atrial fibrillation in the established goat model of AF [156]. Despite these promising preclinical results, the efficacy of PAR1 and PAR2 inhibition in reducing atrial fibrosis requires rigorous evaluation through well-designed clinical trials.

The pro-inflammatory and profibrotic functions of cardiac fibroblasts exhibit distinct sex-specific characteristics [157,158]. In human atrial tissue slices, estrogen administration was shown to downregulate the expression of ACE while upregulating ACE2, which encodes the ACE2 enzyme known to counterbalance ACE activity [157]. Notably, estrogen conferred a protective antifibrotic effect in tissue slices derived from male patients, although the study did not examine its effects on tissue from female patients [157]. Further investigations in murine models revealed that cardiac fibroblasts from male mice expressed higher levels of the pro-inflammatory chemokines CCL2 and CCL7 compared to fibroblasts from female mice [158]. Consequently, hearts from male mice exhibited a heightened pro-inflammatory response. Conversely, hearts from female mice demonstrated an increased presence of anti-inflammatory CD68+CD206+ macrophages [158]. Interestingly, despite these differences, the net outcome was a higher cardiac collagen content in female hearts, consistent with greater atrial fibrosis observed in atrial tissue samples from female patients compared to male patients [16]. These findings highlight the potential for differential responses to antifibrotic therapies between sexes and underscore the importance of exploring sex-specific strategies for antifibrotic treatment development [158].

Several microRNAs (miRNAs) have been identified as inhibitors of gene expression associated with cardiac fibrosis. For instance, miR-29, miR-30, and miR-133 suppress collagen production, while miR-21 downregulates SMAD3 expression, and miR-590 inhibits TGFβ1 expression [159,160,161,162]. Additionally, other miRNAs implicated in atrial fibrosis, such as miR-135b-5p and miR-138-5p, influence glycosaminoglycan biosynthesis [163]. However, further mechanistic validation is required to confirm their roles in fibrosis regulation. Notably, a study demonstrated that transfection of miR-146b-5p into mouse cardiac fibroblasts led to TIMP4 inhibition and enhanced collagen synthesis, indicating complex regulatory dynamics [164]. Beyond miRNAs, epigenetic mechanisms also present promising therapeutic avenues for addressing atrial fibrosis [165]. Histone deacetylases, for example, regulate pathogenic gene expression in AF [164]. Additionally, increased expression of the histone-lysine N-methyltransferase EZH2, which catalyzes histone 3 methylation, has been linked to fibrosis in AF patients [166]. Further exploration of the intricate gene networks involved in atrial fibrosis and AF could yield novel therapeutic targets. Comprehensive reviews on these molecular pathways and their therapeutic potential have been published elsewhere [167].

**Figure 2:**
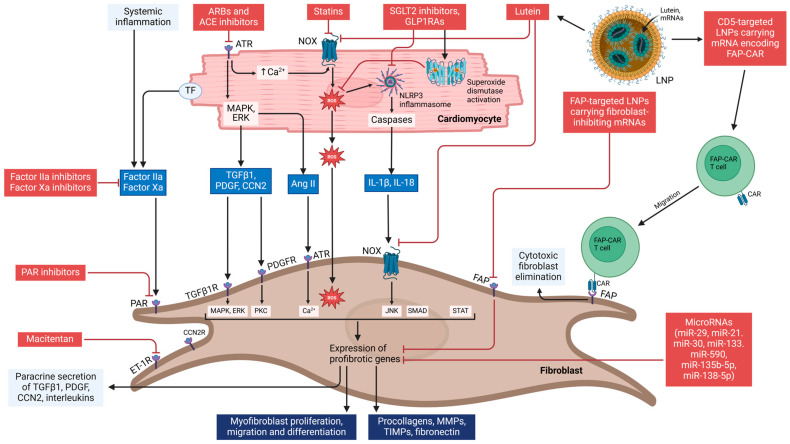
Potential therapeutic targets for the prevention and mitigation of atrial fibrosis.

Lutein demonstrates significant anti-inflammatory, antioxidant, and antifibrotic properties in the context of cardiac injury [168]. However, its therapeutic application is constrained by its low bioavailability and preferential accumulation in ocular tissues [168]. Advances in drug delivery systems, such as lipid nanoparticles (LNPs), which gained widespread clinical use during the COVID-19 pandemic, may offer a more effective and targeted alternative to conventional small-molecule drugs [169]. Notably, macrophage membrane-coated LNPs have been successfully tested, showing high specificity to cardiac tissue with minimal toxicity [169]. Studies have demonstrated that lutein encapsulated in macrophage membrane-coated LNPs mitigates cardiac fibrosis induced by pressure overload and suppresses angiotensin II-driven fibroblast activation by modulating the ERK signaling pathway in murine models [168]. Furthermore, mRNA-loaded LNPs represent a novel therapeutic avenue for addressing cardiac fibrosis [170]. In models of cardiac injury, LNPs have been utilized to deliver fibroblast-specific chimeric antigen receptor (CAR) T cells, which selectively target and eliminate activated fibroblasts, thereby reducing fibrosis [170]. LNPs also hold the potential for the delivery of mRNAs designed to directly inhibit profibrotic pathways, broadening the scope of antifibrotic interventions. However, the cell-specific nature of profibrotic gene expression presents a critical challenge [171]. Inhibiting fibrotic activity in cells not contributing to pathological fibrosis in the atrium could result in unintended adverse effects [171]. A targeted approach focusing on fibroblasts activated specifically in response to tissue injury may help minimize these risks and enhance therapeutic efficacy [170]. The potential therapeutic approaches are summarized in Figure 2.

Early intervention with catheter ablation for AF holds promise in curbing the progression of atrial cardiomyopathy, including the development of atrial fibrosis, provided the procedure is performed in the initial stages of the disease and achieves complete suppression of AF [172]. However, as shown in the DECAAF II trial, strategies guiding catheter ablation based on atrial fibrosis identified through LGE-CMR have proven ineffective [89].

Incorporating insights from recent trials, such as ERASE AF, offers an important perspective on the evolving role of LVA-guided ablation [173]. This trial demonstrated that targeting LVAs identified via high-density mapping significantly reduced AF recurrences compared to conventional PVI [173]. These findings suggest that identifying and targeting fibrotic regions that create an arrhythmogenic substrate may translate into improved survival free from AF recurrences. Such advancements underscore the importance of refining patient selection criteria and integrating low-voltage mapping into standard ablation protocols. Particularly in individuals with advanced atrial fibrosis, this approach aligns with the growing emphasis on personalized, substrate-based therapeutic strategies. The ERASE AF trial provides a framework to address limitations observed in fibrosis-guided ablation strategies, such as those highlighted in the DECAAF II trial, by focusing on improved procedural precision and outcomes.

A complementary perspective is provided by the study conducted by Chelu et al. [174], which assessed the long-term implications of atrial fibrosis on ablation outcomes using LGE-CMR. This study followed 308 patients over a 5-year period and demonstrated a clear association between the degree of left atrial fibrosis and AF ablation success [174]. Patients with advanced fibrosis (Utah stage IV, >30%) experienced significantly higher rates of arrhythmia recurrence and repeat ablation compared to those with minimal fibrosis (Utah stage I, 0–10%) [174]. Specifically, the hazard ratio for recurrence in stage IV versus stage I was 2.73 (95% CI, 1.57–4.75), and the proportional odds ratio for repeat ablation was 5.19 (95% CI, 2.12–12.69) [174].

These findings highlight the prognostic value of LGE-CMR in quantifying atrial fibrosis and predicting long-term ablation outcomes. However, advanced fibrosis remains a strong determinant of procedural failure, underscoring the limitations of current imaging-based strategies in addressing fibrotic remodeling.

Further supporting the role of fibrosis-guided strategies, a systematic review and meta-analysis by Ahn et al. [175] evaluated randomized controlled trials comparing fibrosis-guided ablation, using LVA or LGE-CMR, with PVI. This analysis of 2135 patients demonstrated that fibrosis-guided ablation significantly reduced atrial arrhythmia recurrence (risk ratio 0.82; 95% CI, 0.71–0.94; *p* = 0.006) without increasing procedural time, fluoroscopic time, or adverse events [175]. Stratified analyses showed consistent efficacy across fibrosis identification methods, AF type, and ablation strategies [175].

Despite these promising results, the absence of significant subgroup interactions in the meta-analysis suggests that fibrosis-guided approaches require further refinement to optimize patient selection and treatment strategies. Together, these findings emphasize the need for novel methods to more effectively address fibrotic remodeling in AF management.

Atrial fibrosis is driven by complex paracrine signaling networks regulating fibroblast proliferation, activation, and collagen synthesis. Therapeutic strategies target key pathways, including the renin–angiotensin–aldosterone system (RAAS), endothelin-1 (ET-1) signaling, protease-activated receptors, and microRNAs (miRNAs). Pharmacological agents such as angiotensin-converting enzyme (ACE) inhibitors, angiotensin II receptor blockers (ARBs), macitentan, and nadroparin show potential in experimental models. Innovations like lipid nanoparticles (LNPs) and chimeric antigen receptor (CAR) T cells enable targeted delivery of antifibrotic agents. Emerging antidiabetic drugs, including sodium-glucose co-transporter 2 (SGLT2) inhibitors and glucagon-like peptide-1 receptor agonists (GLP-1RAs), reduce oxidative stress and pro-fibrotic signaling. These integrated approaches offer promising strategies to address the multifaceted drivers of atrial fibrosis.

### Knowledge Gaps and Future Directions

Despite significant advances in understanding the pathophysiological role of atrial fibrosis in AF, several critical knowledge gaps persist. First, the precise mechanisms by which different types of fibrosis—such as reactive and replacement fibrosis—affect atrial conduction and arrhythmogenesis remain incompletely understood. A clearer distinction between these mechanisms is essential to tailor therapeutic approaches.

Current imaging techniques, such as LGE-CMR, lack standardization and spatial resolution sufficient to reliably quantify the extent and type of atrial fibrosis. This limitation hampers the development of fibrosis-targeted strategies and their validation in clinical settings. Similarly, while electroanatomical mapping provides surrogate markers like LVAs, these measures correlate inconsistently with histological findings and lack specificity for fibrosis.

Emerging biomarkers, such as BMP10 and various microRNAs, show potential for non-invasive fibrosis assessment, yet their specificity and reproducibility in broader populations require further validation. Additionally, the interplay between sex-specific factors, comorbidities, and fibrosis development is underexplored and warrants focused investigation to optimize individualized treatment strategies.

Therapeutically, while promising antifibrotic agents—including SGLT2 inhibitors and GLP-1RAs—target known profibrotic pathways, their long-term impact on atrial fibrosis and clinical outcomes remains unclear. Novel approaches, such as lipid nanoparticle-based delivery systems and fibroblast-specific CAR T cells, represent exciting avenues but are still in early preclinical stages and face translational hurdles.

To address these challenges, future research should focus on the following key areas:

1. Improved Diagnostics: Develop and standardize high-resolution imaging modalities and integrate molecular biomarkers for accurate, reproducible fibrosis quantification.
2. Mechanistic Insights: Conduct studies to delineate the molecular drivers of different fibrosis types and their specific impacts on atrial conduction.
3. Therapeutic Validation: Design large-scale, multicenter clinical trials to evaluate the efficacy of emerging antifibrotic therapies in reducing fibrosis and improving patient outcomes.
4. Innovative Models: Employ advanced platforms, such as human-induced pluripotent stem cell-derived atrial tissues and precision animal models, to study fibrosis mechanisms and test interventions in a controlled yet translationally relevant manner.
5. Personalized Medicine: Investigate how sex-specific differences, genetic predispositions, and comorbidities influence fibrosis progression and therapeutic responses to optimize treatment personalization.

Bridging these knowledge gaps will require collaborative efforts across basic, translational, and clinical research domains.

Moreover, recent studies highlight that, in addition to fibrosis, atrial amyloidosis significantly contributes to atrial arrhythmogenesis and thrombotic risk [176]. The infiltrative nature of amyloid deposits within atrial tissue disrupts normal electrophysiological and structural integrity, fostering an environment conducive to arrhythmias such as AF and increasing susceptibility to thromboembolic events. Casella et al. [176] demonstrated that electroanatomic abnormalities, including low-voltage zones and dense scar areas, are not only prevalent in cardiac amyloidosis but also correlate with amyloid burden and replacement fibrosis, serving as potential predictors of clinical outcomes, including adverse thrombotic events [176]. These findings underscore the importance of recognizing atrial amyloidosis as a key player in atrial pathology beyond fibrosis, warranting further exploration of its mechanistic roles in arrhythmogenesis and thrombosis.

## 5. Conclusions

Atrial fibrosis plays a central role in the pathogenesis and progression of AF by creating electrical conduction heterogeneities and structural remodeling. It is driven by complex interactions involving fibroblasts, cardiomyocytes, and immune cells. These changes, particularly in the subepicardial layer, contribute to arrhythmogenic mechanisms that sustain AF. Despite advances in imaging, biomarkers, and mechanistic understanding, challenges in accurately quantifying fibrosis and its direct clinical impacts remain.

Future efforts should prioritize refining diagnostic tools, such as integrating molecular and imaging biomarkers, to enhance the precision of fibrosis assessment. Additionally, therapeutic interventions, including antifibrotic agents, microRNA-based treatments, and innovative technologies like lipid nanoparticle delivery systems, hold promise for addressing the underlying fibrotic processes. However, large-scale, randomized trials are necessary to validate these strategies and evaluate their efficacy in improving clinical outcomes. Achieving this goal will require not only the identification of actionable fibrotic pathways but also the development of reliable and reproducible tools for quantifying atrial fibrosis in clinical settings.

By bridging knowledge gaps through rigorous research and leveraging emerging technologies, a comprehensive approach to mitigating atrial fibrosis and its complications in AF can be achieved, ultimately improving patient care and outcomes.

## Figures and Tables

**Table 1:** Studies investigating the impact of atrial fibrosis on electrical conduction.

Study, Year	Study Type	Model/Population	Main Findings	Implications
Li et al., 1999 [45]	Preclinical	Canine models with RAP and CHF	CHF induced by rapid ventricular pacing significantly increased AF duration (535 ± 82 s) without altering atrial refractoriness, unlike RAP, which primarily affected the refractory period. HF caused substantial conduction heterogeneity during atrial pacing and was associated with extensive interstitial fibrosis (12.8 ± 1.9% vs. 0.8 ± 0.3% in controls).	CHF-related AF is driven by interstitial fibrosis disrupting local conduction, representing a distinct substrate compared to RAP-induced AF. Targeting fibrosis may be critical for preventing and managing AF associated with CHF, emphasizing the need for substrate-specific therapeutic strategies.
Shinagawa et al., 2002 [49]	Preclinical	Canine models recovering from CHF	CHF-induced AF duration and atrial enlargement improved after recovery, but atrial fibrosis (10.7 ± 1.0% in CHF vs. 3.1 ± 0.3% in controls) and conduction heterogeneity persisted (2.3 ± 0.1 in CHF vs. 1.8 ± 0.1 in controls). Despite hemodynamic normalization, sustained AF could still be induced due to irreversible structural remodeling, including fibrosis and conduction abnormalities.	Structural changes like atrial fibrosis are irreversible, even after CHF resolution, and contribute to a persistent AF substrate. Early intervention targeting structural remodeling is crucial to prevent irreversible changes and reduce long-term AF risk.
Ausma et al., 2001 [53]	Preclinical	Goat model with sustained AF	In goats with AF, cellular structural remodeling developed progressively, with initial chromatin changes at 1 week and increasing myolysis and glycogen accumulation until 8 weeks (affecting 42% of myocytes by 16 weeks). Dedifferentiation was indicated by altered expression of structural proteins, including loss of cardiotin (1 week), titin (4 weeks), and desmin (8 weeks), with gradual re-expression of alpha-smooth muscle actin.	Progressive cellular remodeling and dedifferentiation during AF suggest a shift toward a fetal-like phenotype, contributing to atrial dysfunction. Understanding these changes could inform strategies to prevent or reverse structural remodeling in AF, improving myocardial integrity and function.
Ravelli et al., 2023 [55]	Clinical	Human atrial tissue from cardiac surgery patients	Intramural fibrosis progressively decreased from 68.6 ± 11.6% in the subepicardium to 10–13% in the subendocardium, with slower fibrosis decay in patients with atrial dilatation (171.2 ± 54.5 µm) or AF (142.8 ± 41.7 µm) compared to controls (80.9 ± 24.4 µm). Subepicardial and midwall fibrosis correlated strongly with atrial dilatation (ρ = 0.72, *p* < 0.001), while subendocardial fibrosis showed no such correlation.	Deeper penetration of fibrosis into subepicardial and midwall layers in dilated atria contributes to a 3D substrate for AF, emphasizing the importance of regional fibrosis assessment. High-resolution histological quantification provides insights into the structural remodeling underlying AF, potentially guiding targeted interventions.
Hansen et al., 2015 [74]	Clinical	Human hearts (explanted)	Sustained AF was driven by intramural re-entry anchored to micro-anatomic tracks formed by fibrosis-insulated atrial bundles, with re-entrant drivers primarily visualized on sub-endo mapping.	These findings highlight the role of atrial microstructural complexity in creating substrates for sustained AF, emphasizing the importance of high-resolution Endo-Epi mapping in identifying AF drivers. The identification of fibrosis-anchored re-entry as AF drivers suggests that targeted ablation of these tracks could improve therapeutic strategies for AF management.
Winters et al., 2023 [16]	Clinical	Human left atrium	Persistent AF and HF were associated with increased endomysial fibrosis and ECM content in fibrotic atCM, while cardiomyocyte hypertrophy was the hallmark of hypertrophic atCM. Fibrotic atCM was more common in women and linked to persistent AF and HF, whereas hypertrophic atCM was more frequent in men.	These findings highlight the impact of sex and clinical conditions like AF and HF on distinct atCM phenotypes, supporting the need for individualized diagnostic and therapeutic strategies. Differentiating fibrotic and hypertrophic atCM subtypes provides critical insights into atrial remodeling and may guide precision medicine in CVD.
Ramos et al., 2022 [70]	Clinical	Human atrial tissue	The degree of fibrosis in atrial tissue and serum did not differ significantly between controls and patients at various stages of AF. No correlation, absolute or spatial, was found between electrophysiological abnormalities and histological fibrosis markers.	These findings challenge the traditional view of fibrosis as the primary driver of AF-related structural remodeling, suggesting the need to investigate alternative mechanisms. A lack of correlation between fibrosis and electrophysiological abnormalities underscores the complexity of AF pathophysiology and calls for a broader focus beyond fibrosis in AF research and treatment.
Krul et al., 2015 [73]	Clinical	Left atrial appendage tissue from surgical patients	Thick interstitial collagen strands in the left atrial appendage (LAA) were associated with higher longitudinal conduction velocity (CVL, 0.77 ± 0.22 vs. 0.48 ± 0.19 m/s, *p* = 0.012) and prolonged activation times (14.93 ± 4.12 vs. 7.95 ± 4.12 ms, *p* = 0.004). Fibroblasts were abundant and linked to thick collagen strands, while no myofibroblasts were detected, and fibrosis severity did not correlate with transverse conduction velocity (CVT) or patient characteristics.	The structural organization of interstitial fibrosis, particularly the presence of thick collagen strands, significantly affects atrial conduction, creating a substrate for arrhythmogenic re-entry in AF. These findings suggest that targeting the structural characteristics of fibrosis beyond its quantity could refine strategies for managing conduction abnormalities in AF.
Gharaviri et al., 2020 [57]	Clinical	Human atrial tissue with imaging and mapping	Increased epicardial fibrosis led to a significant rise in endo-epicardial dissociation (EED, 24.1 ± 3.4% to 56.58 ± 6.2%, *p* < 0.05) and breakthroughs (BTs, 0.89 ± 0.55 to 6.74 ± 2.11 per cycle, *p* < 0.05) in a 3D human atrial model, with similar results observed in patient mapping data. Epicardial fibrosis also increased the number of fibrillation waves per cycle, correlating with higher EED and BT prevalence, even in the absence of other pathological changes.	These findings establish epicardial fibrosis as a direct cause of EED and BT, demonstrating its key role in perpetuating arrhythmogenic activity in persistent AF. Targeting epicardial fibrosis may provide a novel therapeutic avenue for disrupting the mechanisms underlying EED and BT in AF.
Lee et al., 2015 [76]	Clinical	High-density mapping in patients	Persistent and LSP AF were sustained by multiple focal sources (2–4 per patient, duration 5–32 s) and breakthrough activation sites, primarily in the lateral left atrial free wall, rather than by reentrant circuits. Wavefronts from these foci and breakthroughs propagated throughout the atria, often colliding or merging, and occasionally mimicked reentrant patterns, but no true reentry was observed.	AF maintenance in persistent and LSP AF is predominantly driven by focal and breakthrough activity, challenging the role of reentry as a primary mechanism. Therapies targeting focal sources and breakthrough sites, particularly in the left atrial free wall, may offer improved outcomes for patients with persistent or LSP AF.

Abbreviations: AF, atrial fibrillation; LSP, long-standing persistent; EED, endo-epicardial dissociation; BT, breakthroughs; CV, conduction velocity; CVL, longitudinal conduction velocity; CVT, transverse conduction velocity; ECM, extracellular matrix; H&E, hematoxylin and eosin; BMP10, bone morphogenetic protein 10; NT-pro-BNP, N-terminal pro b-type natriuretic peptide; RA, right atrium; LA, left atrium; LGE-CMR, late gadolinium enhancement cardiac magnetic resonance; LVAs, low-voltage areas; RAP, rapid atrial pacing; CHF, congestive heart failure.

**Table 2:** Studies evaluating imaging methods for diagnosing and characterizing atrial fibrosis.

Study, Year	Setting/Population	Imaging Modality	Main Findings	Implications
Gunturiz-Beltrán et al., 2023 [83]	53 patients with AF	LGE-CMR	An image intensity ratio (IIR) > 1.21 identifies total right atrial (RA) fibrosis, while an IIR > 1.29 distinguishes interstitial fibrosis from dense scar, with a weak but significant correlation to bipolar voltage. These thresholds align closely with those used for LA fibrosis assessment, highlighting reproducibility across chambers.	LGE-CMR offers a standardized, noninvasive tool for assessing atrial remodeling. This facilitates comprehensive bi-atrial characterization in AF patients, potentially improving personalized diagnostic and therapeutic strategies.
Eichenlaub et al., 2022 [82]	37 ablation-naïve patients with persistent AF	LGE-CMR	Significant discrepancies were observed in ACM extent and distribution between various LGE-MRI methods and voltage mapping. LVS > 2 cm^2^ at 0.5 mV strongly predicted arrhythmia recurrence after PVI, while no correlation was found between LGE-detected fibrosis and conduction slowing or recurrence.	Current LGE-MRI protocols require standardization and refinement to reliably diagnose ACM and predict PVI outcomes, underlining the superior prognostic value of LVS mapping in persistent AF patients.
Verheule et al., 2013 [42]	Patients with AF	Electroanatomical catheter mapping	In a goat model of AF, LT AF showed a more homogeneous distribution of wave origins and conduction abnormalities compared to ST AF. This was associated with increased endomysial fibrosis, particularly in the atrial epicardium, leading to slower, more anisotropic wavefront propagation and greater fibrillation complexity.	The distribution and type of fibrosis, particularly in the epicardial layer, play a key role in altering conduction pathways and increasing AF complexity, highlighting the importance of targeting specific structural changes rather than overall fibrosis quantity for effective interventions.
Zahid et al., 2016 [88]	20 patients with AF	LGE-CMR, Computational modeling	Patient-derived models revealed that AF in fibrotic atria is sustained by re-entrant drivers confined to fibrotic boundary zones with high fibrosis density and entropy. These zones, comprising only ~14% of atrial tissue, accounted for ~84% of re-entrant driver activity, highlighting their critical role in persistent AF.	Identifying RD-prone zones using MRI-derived fibrosis metrics could enable personalized strategies for AF management, targeting regions sustaining AF for improved outcomes.
Hansen et al., 2015 [74]	Explanted human atrial tissue	Optical mapping	Sustained AF was driven by intramural re-entrant circuits anchored to fibrosis-insulated muscle bundles with distinct transmural fiber angles and structural heterogeneity. These re-entrant drivers, primarily visualized via sub-endo mapping, exhibited stable activation patterns with significant transmural delays.	The study highlights the role of 3D microstructural features in sustaining AF and the value of combined structural–functional mapping for identifying and targeting specific atrial regions for effective ablation therapy.
Marrouche et al., 2022 [89]	843 patients in the DECAAF II trial	LGE-CMR-guided ablation	In patients with persistent AF, MRI-guided fibrosis ablation plus PVI did not significantly reduce atrial arrhythmia recurrence compared to PVI alone (43.0% vs. 46.1%). However, the fibrosis-guided group had a higher rate of adverse events, including ischemic stroke and two deaths.	MRI-guided fibrosis ablation does not improve outcomes over standard PVI in persistent AF and is associated with increased procedural risks, questioning its utility in clinical practice.

Abbreviations: AF, atrial fibrillation; LGE-CMR, late gadolinium enhancement cardiovascular magnetic resonance; IIR, image intensity ratio; RA, right atrium; LA, left atrium; ACM, atrial cardiomyopathy; LVS, low-voltage substrate; PVI, pulmonary vein isolation.

## Data Availability

All data generated in this research are included within the article.

## Abbreviations

CCN2—CCN family member 2; CCN2R—CCN family member 2 receptor; ERK—extracellular signal-regulated kinase; FAP—fibroblast-activating protein; JNK—JUN amino-terminal kinase; MAPK—mitogen-activated protein kinase; MMPs—matrix metalloproteinases; NOX—NADPH oxidases; PDGF—platelet-derived growth factor; PDGFR—platelet-derived growth factor receptor; PKC—protein kinase C; ROS—reactive oxygen species; STAT—signal transducers and activators of transcription; TF—tissue factor; TGFβ1R—transforming growth factor-β1 receptor; TIMPs—tissue inhibitors of metalloproteinases.
